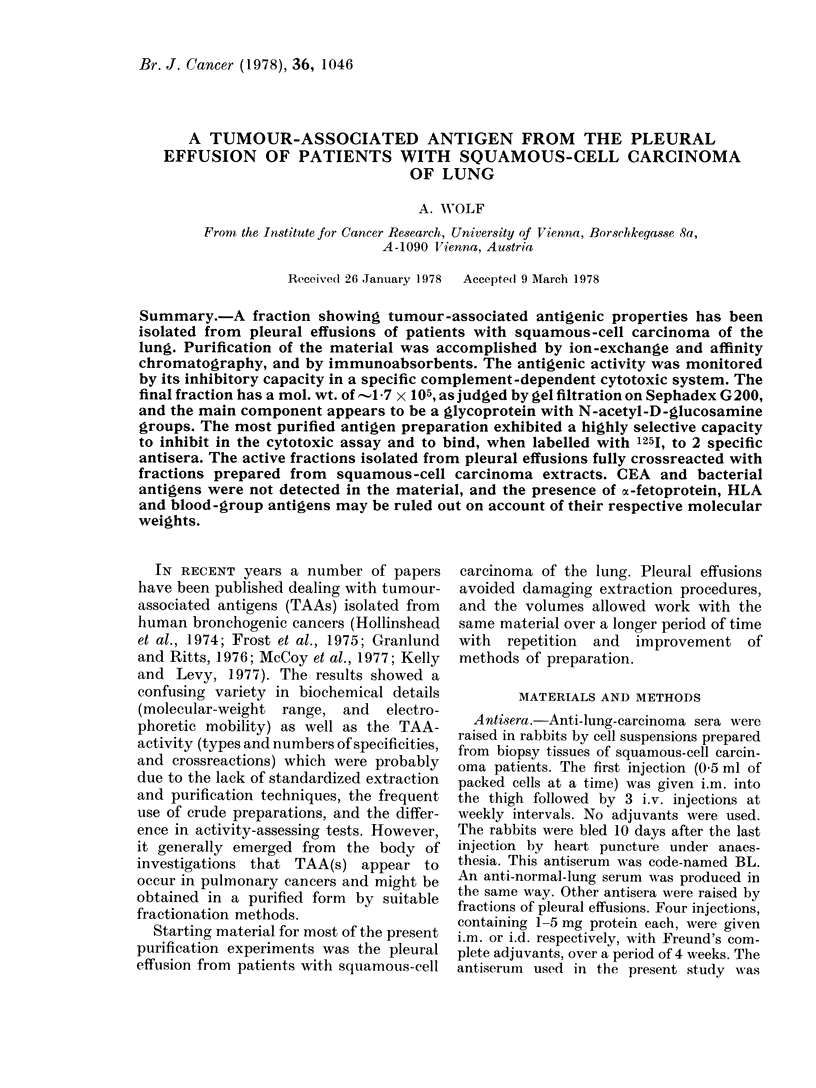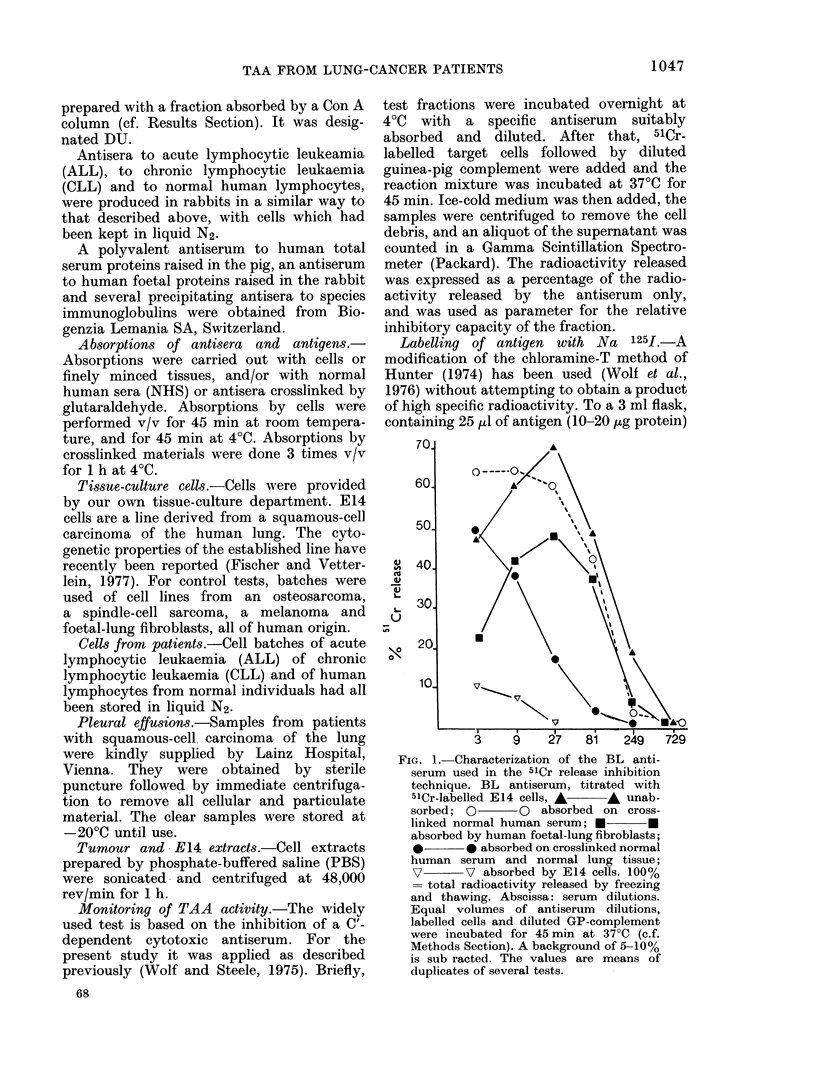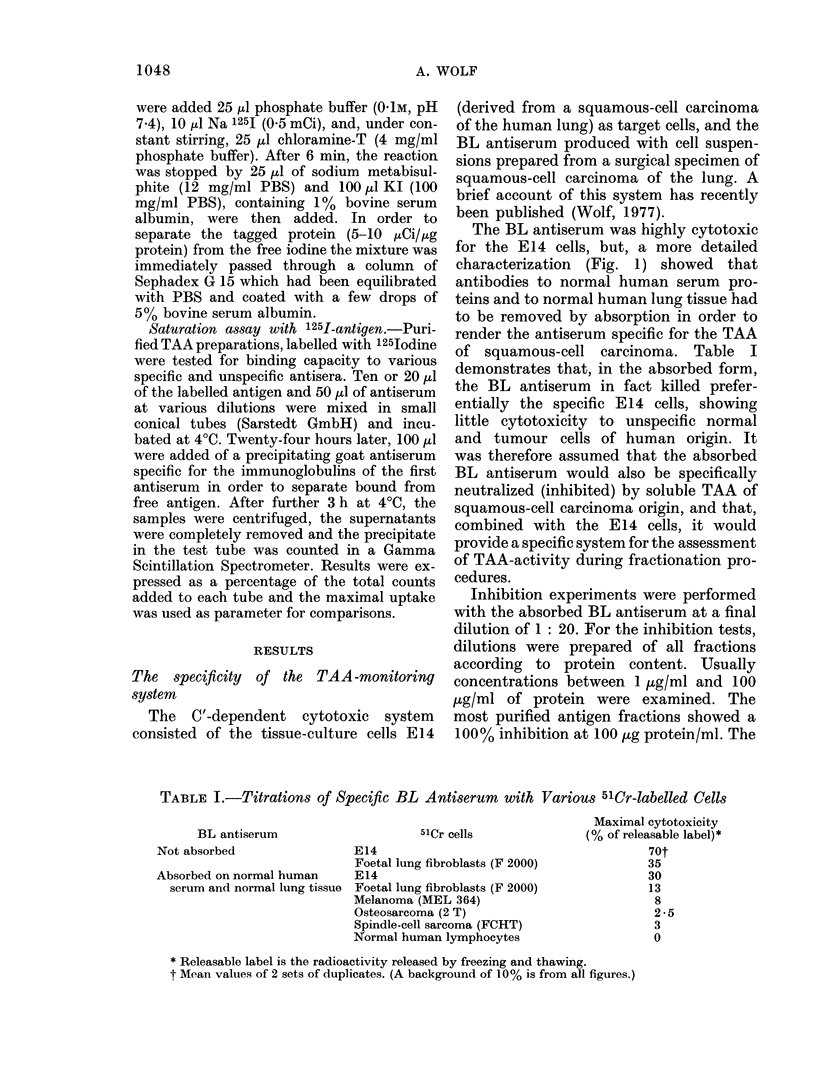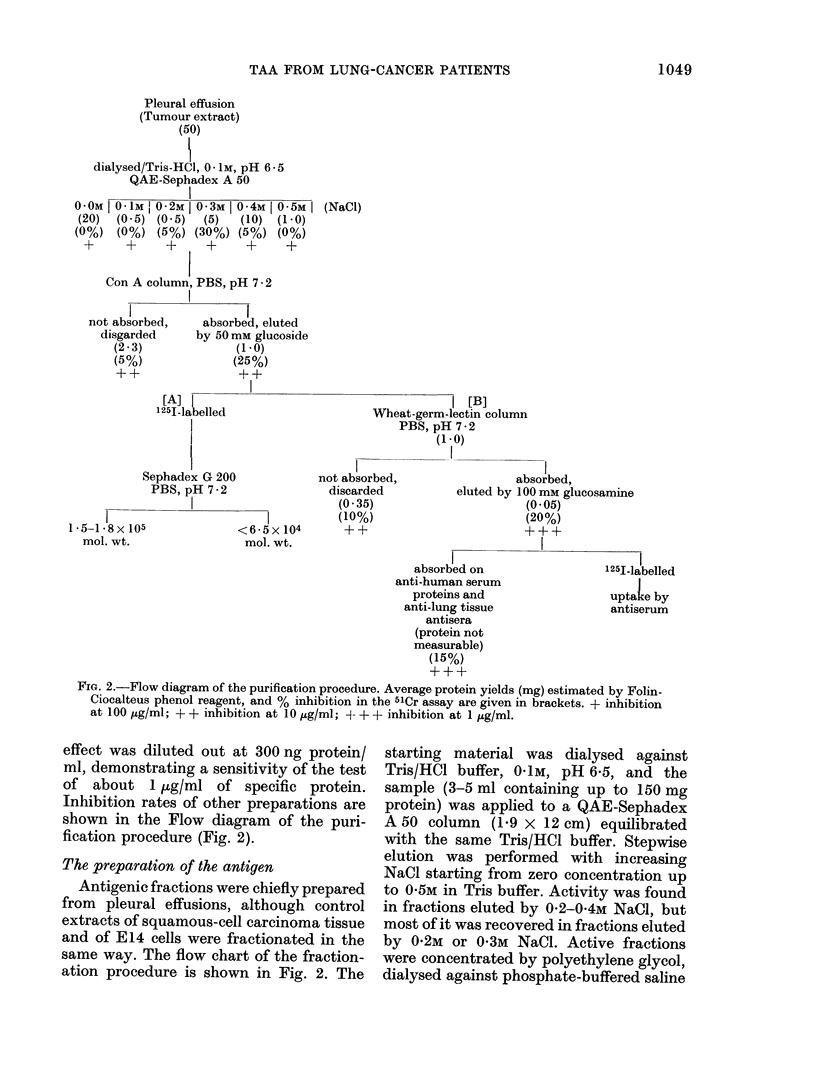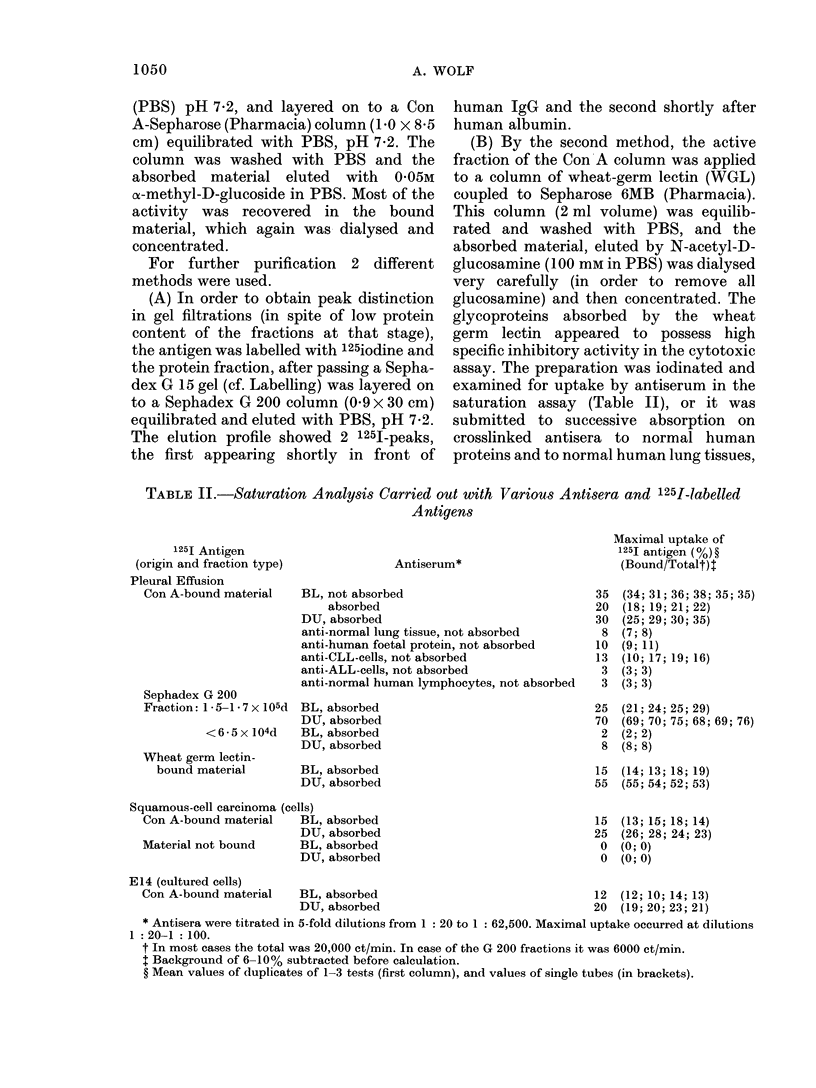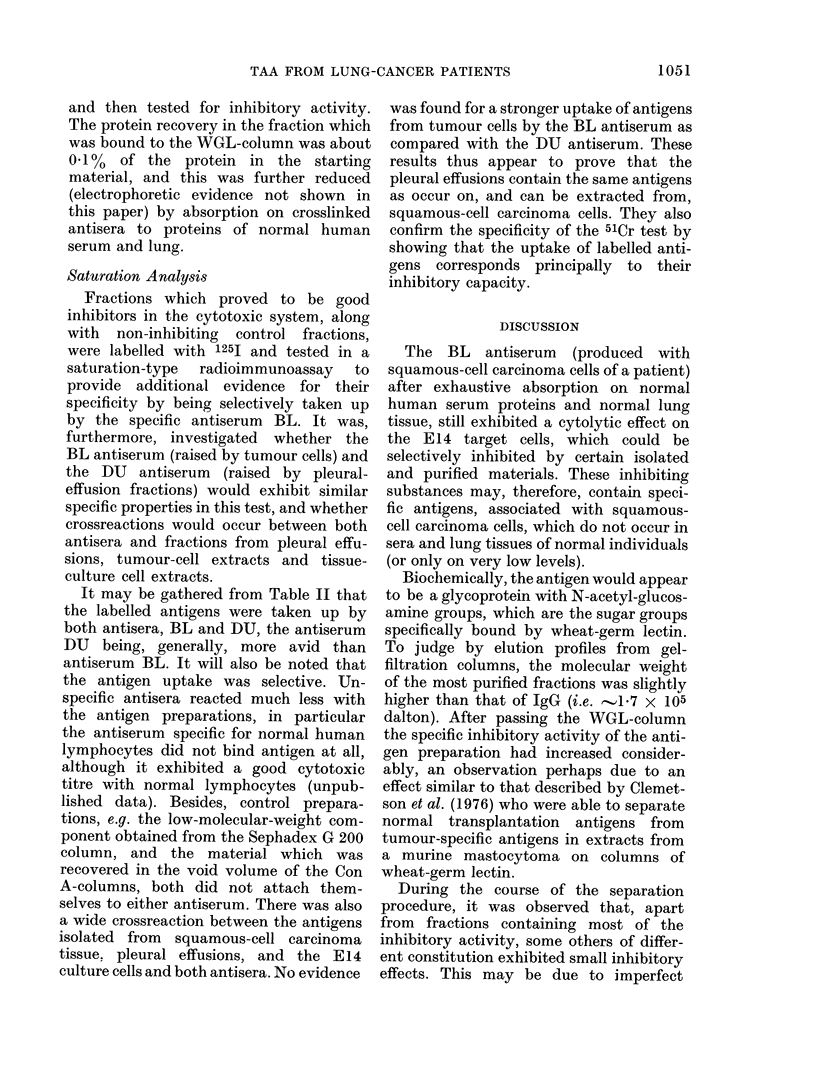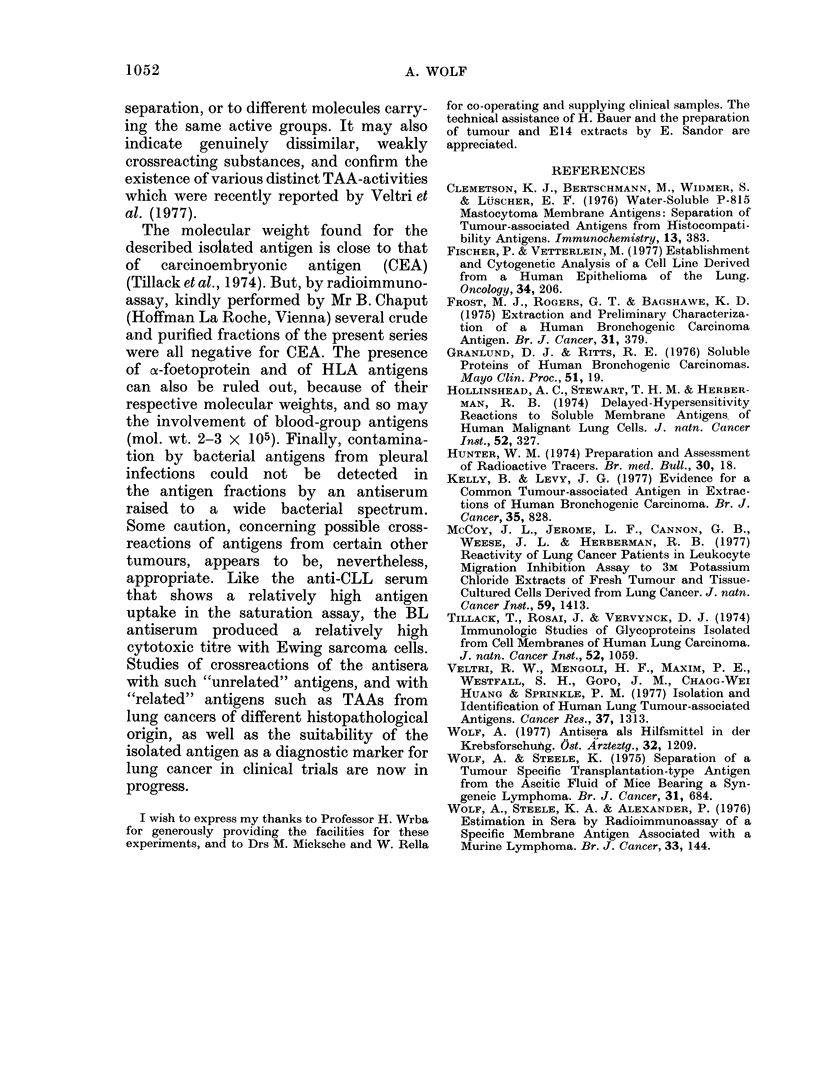# A tumour-associated antigen from the pleural effusion of patients with squamous-cell carcinoma of lung.

**DOI:** 10.1038/bjc.1978.152

**Published:** 1978-06

**Authors:** A. Wolf

## Abstract

A fraction showing tumour-associated antigenic properties has been isolated from pleural effusions of patients with squamous-cell carcinoma of the lung. Purification of the material was accomplished by ion-exchange and affinity chromatography, and by immunoabsorbents. The antigenic activity was monitored by its inhibitory capacity in a specific complement-dependent cytotoxic system. The final fraction has a mol. wt. of approximately 1.7 X 10(5), as judged by gel filtration on Sephadex G200, and the main component appears to be a glycoprotein with N-acetyl-D-glucosamine groups. The most purified antigen preparation exhibited a highly selective capacity to inhibit in the cytotoxic assay and to bind, when labelled with 125I, to 2 specific antisera. The active fractions isolated from pleural effusions fully crossreacted with fractions prepared from squamous-cell carcinoma extracts. CEA and bacterial antigens were not detected in the material, and the presence of alpha-fetoprotein, HLA and blood-group antigens may be ruled out on account of their respective molecular weights.


*Br. J. Cancer* (1978), **36**, 1046

### A TUMOUR-ASSOCIATED ANTIGEN FROM THE PLEURAL EFFUSION OF PATIENTS WITH SQUAMOUS-CELL CARCINOMA OF LUNG

A. WOLF

*From the Institute for Cancer Research, University of Vienna, Borschkegasse 8a, A-1090 Vienna, Austria*

Received 26 January 1978 Accepted 9 March 1978

**Summary.—A fraction showing tumour-associated antigenic properties has been isolated from pleural effusions of patients with squamous-cell carcinoma of the lung. Purification of the material was accomplished by ion-exchange and affinity chromatography, and by immunoabsorbents. The antigenic activity was monitored by its inhibitory capacity in a specific complement-dependent cytotoxic system. The final fraction has a mol. wt. of $\sim 1 \cdot 7 \times 10^5$, as judged by gel filtration on Sephadex G200, and the main component appears to be a glycoprotein with N-acetyl-D-glucosamine groups. The most purified antigen preparation exhibited a highly selective capacity to inhibit in the cytotoxic assay and to bind, when labelled with $^{125}I$, to 2 specific antisera. The active fractions isolated from pleural effusions fully crossreacted with fractions prepared from squamous-cell carcinoma extracts. CEA and bacterial antigens were not detected in the material, and the presence of $\alpha$-fetoprotein, HLA and blood-group antigens may be ruled out on account of their respective molecular weights.**

IN RECENT years a number of papers have been published dealing with tumour-associated antigens (TAAs) isolated from human bronchogenic cancers (Hollinshead *et al.*, 1974; Frost *et al.*, 1975; Granlund and Ritts, 1976; McCoy *et al.*, 1977; Kelly and Levy, 1977). The results showed a confusing variety in biochemical details (molecular-weight range, and electrophoretic mobility) as well as the TAA-activity (types and numbers of specificities, and crossreactions) which were probably due to the lack of standardized extraction and purification techniques, the frequent use of crude preparations, and the difference in activity-assessing tests. However, it generally emerged from the body of investigations that TAA(s) appear to occur in pulmonary cancers and might be obtained in a purified form by suitable fractionation methods.

Starting material for most of the present purification experiments was the pleural effusion from patients with squamous-cell carcinoma of the lung. Pleural effusions avoided damaging extraction procedures, and the volumes allowed work with the same material over a longer period of time with repetition and improvement of methods of preparation.

### MATERIALS AND METHODS

*Antisera.*—Anti-lung-carcinoma sera were raised in rabbits by cell suspensions prepared from biopsy tissues of squamous-cell carcinoma patients. The first injection (0·5 ml of packed cells at a time) was given i.m. into the thigh followed by 3 i.v. injections at weekly intervals. No adjuvants were used. The rabbits were bled 10 days after the last injection by heart puncture under anaesthesia. This antiserum was code-named BL. An anti-normal-lung serum was produced in the same way. Other antisera were raised by fractions of pleural effusions. Four injections, containing 1–5 mg protein each, were given i.m. or i.d. respectively, with Freund's complete adjuvants, over a period of 4 weeks. The antiserum used in the present study was prepared with a fraction absorbed by a Con A column (cf. Results Section). It was designated DU.

Antisera to acute lymphocytic leukeamia (ALL), to chronic lymphocytic leukaemia (CLL) and to normal human lymphocytes, were produced in rabbits in a similar way to that described above, with cells which had been kept in liquid $N_2$.

A polyvalent antiserum to human total serum proteins raised in the pig, an antiserum to human foetal proteins raised in the rabbit and several precipitating antisera to species immunoglobulins were obtained from Biogenzia Lemania SA, Switzerland.

*Absorptions of antisera and antigens.*—Absorptions were carried out with cells or finely minced tissues, and/or with normal human sera (NHS) or antisera crosslinked by glutaraldehyde. Absorptions by cells were performed v/v for 45 min at room temperature, and for 45 min at 4°C. Absorptions by crosslinked materials were done 3 times v/v for 1 h at 4°C.

*Tissue-culture cells.*—Cells were provided by our own tissue-culture department. E14 cells are a line derived from a squamous-cell carcinoma of the human lung. The cytogenetic properties of the established line have recently been reported (Fischer and Vetterlein, 1977). For control tests, batches were used of cell lines from an osteosarcoma, a spindle-cell sarcoma, a melanoma and foetal-lung fibroblasts, all of human origin.

*Cells from patients.*—Cell batches of acute lymphocytic leukaemia (ALL) of chronic lymphocytic leukaemia (CLL) and of human lymphocytes from normal individuals had all been stored in liquid $N_2$.

*Pleural effusions.*—Samples from patients with squamous-cell carcinoma of the lung were kindly supplied by Lainz Hospital, Vienna. They were obtained by sterile puncture followed by immediate centrifugation to remove all cellular and particulate material. The clear samples were stored at −20°C until use.

*Tumour and E14 extracts.*—Cell extracts prepared by phosphate-buffered saline (PBS) were sonicated and centrifuged at 48,000 rev/min for 1 h.

*Monitoring of TAA activity.*—The widely used test is based on the inhibition of a C′-dependent cytotoxic antiserum. For the present study it was applied as described previously (Wolf and Steele, 1975). Briefly, test fractions were incubated overnight at 4°C with a specific antiserum suitably absorbed and diluted. After that, $^{51}Cr$-labelled target cells followed by diluted guinea-pig complement were added and the reaction mixture was incubated at 37°C for 45 min. Ice-cold medium was then added, the samples were centrifuged to remove the cell debris, and an aliquot of the supernatant was counted in a Gamma Scintillation Spectrometer (Packard). The radioactivity released was expressed as a percentage of the radioactivity released by the antiserum only, and was used as parameter for the relative inhibitory capacity of the fraction.

*Labelling of antigen with Na $^{125}I$.*—A modification of the chloramine-T method of Hunter (1974) has been used (Wolf *et al.*, 1976) without attempting to obtain a product of high specific radioactivity. To a 3 ml flask, containing 25 μl of antigen (10–20 μg protein)

FIG. 1.—Characterization of the BL antiserum used in the $^{51}Cr$ release inhibition technique. BL antiserum, titrated with $^{51}Cr$-labelled E14 cells, ▲——▲ unabsorbed; ○——○ absorbed on crosslinked normal human serum; ■——■ absorbed by human foetal-lung fibroblasts; ●——● absorbed on crosslinked normal human serum and normal lung tissue; ▽——▽ absorbed by E14 cells. 100% = total radioactivity released by freezing and thawing. Abscissa: serum dilutions. Equal volumes of antiserum dilutions, labelled cells and diluted GP-complement were incubated for 45 min at 37°C (c.f. Methods Section). A background of 5–10% is sub racted. The values are means of duplicates of several tests.

were added 25 $\mu$l phosphate buffer (0·1M, pH 7·4), 10 $\mu$l Na $^{125}$I (0·5 mCi), and, under constant stirring, 25 $\mu$l chloramine-T (4 mg/ml phosphate buffer). After 6 min, the reaction was stopped by 25 $\mu$l of sodium metabisulphite (12 mg/ml PBS) and 100 $\mu$l KI (100 mg/ml PBS), containing 1% bovine serum albumin, were then added. In order to separate the tagged protein (5–10 $\mu$Ci/$\mu$g protein) from the free iodine the mixture was immediately passed through a column of Sephadex G 15 which had been equilibrated with PBS and coated with a few drops of 5% bovine serum albumin.

*Saturation assay with $^{125}$I-antigen.*—Purified TAA preparations, labelled with $^{125}$Iodine were tested for binding capacity to various specific and unspecific antisera. Ten or 20 $\mu$l of the labelled antigen and 50 $\mu$l of antiserum at various dilutions were mixed in small conical tubes (Sarstedt GmbH) and incubated at 4°C. Twenty-four hours later, 100 $\mu$l were added of a precipitating goat antiserum specific for the immunoglobulins of the first antiserum in order to separate bound from free antigen. After further 3 h at 4°C, the samples were centrifuged, the supernatants were completely removed and the precipitate in the test tube was counted in a Gamma Scintillation Spectrometer. Results were expressed as a percentage of the total counts added to each tube and the maximal uptake was used as parameter for comparisons.

### RESULTS

### *The specificity of the TAA-monitoring system*

The C′-dependent cytotoxic system consisted of the tissue-culture cells E14 (derived from a squamous-cell carcinoma of the human lung) as target cells, and the BL antiserum produced with cell suspensions prepared from a surgical specimen of squamous-cell carcinoma of the lung. A brief account of this system has recently been published (Wolf, 1977).

The BL antiserum was highly cytotoxic for the E14 cells, but, a more detailed characterization (Fig. 1) showed that antibodies to normal human serum proteins and to normal human lung tissue had to be removed by absorption in order to render the antiserum specific for the TAA of squamous-cell carcinoma. Table I demonstrates that, in the absorbed form, the BL antiserum in fact killed preferentially the specific E14 cells, showing little cytotoxicity to unspecific normal and tumour cells of human origin. It was therefore assumed that the absorbed BL antiserum would also be specifically neutralized (inhibited) by soluble TAA of squamous-cell carcinoma origin, and that, combined with the E14 cells, it would provide a specific system for the assessment of TAA-activity during fractionation procedures.

Inhibition experiments were performed with the absorbed BL antiserum at a final dilution of 1 : 20. For the inhibition tests, dilutions were prepared of all fractions according to protein content. Usually concentrations between 1 $\mu$g/ml and 100 $\mu$g/ml of protein were examined. The most purified antigen fractions showed a 100% inhibition at 100 $\mu$g protein/ml. The

TABLE I.—*Titrations of Specific BL Antiserum with Various $^{51}$Cr-labelled Cells*

| BL antiserum | $^{51}$Cr cells | Maximal cytotoxicity (% of releasable label)* |
|---|---|---|
| Not absorbed | E14 | 70† |
| | Foetal lung fibroblasts (F 2000) | 35 |
| Absorbed on normal human serum and normal lung tissue | E14 | 30 |
| | Foetal lung fibroblasts (F 2000) | 13 |
| | Melanoma (MEL 364) | 8 |
| | Osteosarcoma (2 T) | 2·5 |
| | Spindle-cell sarcoma (FCHT) | 3 |
| | Normal human lymphocytes | 0 |

* Releasable label is the radioactivity released by freezing and thawing.
† Mean values of 2 sets of duplicates. (A background of 10% is from all figures.)

FIG. 2.—Flow diagram of the purification procedure. Average protein yields (mg) estimated by Folin-Ciocalteus phenol reagent, and % inhibition in the $^{51}Cr$ assay are given in brackets. + inhibition at 100 $\mu$g/ml; ++ inhibition at 10 $\mu$g/ml; +++ inhibition at 1 $\mu$g/ml.

effect was diluted out at 300 ng protein/ml, demonstrating a sensitivity of the test of about 1 $\mu$g/ml of specific protein. Inhibition rates of other preparations are shown in the Flow diagram of the purification procedure (Fig. 2).

*The preparation of the antigen*

Antigenic fractions were chiefly prepared from pleural effusions, although control extracts of squamous-cell carcinoma tissue and of E14 cells were fractionated in the same way. The flow chart of the fractionation procedure is shown in Fig. 2. The starting material was dialysed against Tris/HCl buffer, 0·1M, pH 6·5, and the sample (3–5 ml containing up to 150 mg protein) was applied to a QAE-Sephadex A 50 column (1·9 × 12 cm) equilibrated with the same Tris/HCl buffer. Stepwise elution was performed with increasing NaCl starting from zero concentration up to 0·5M in Tris buffer. Activity was found in fractions eluted by 0·2–0·4M NaCl, but most of it was recovered in fractions eluted by 0·2M or 0·3M NaCl. Active fractions were concentrated by polyethylene glycol, dialysed against phosphate-buffered saline

(PBS) pH 7·2, and layered on to a Con A-Sepharose (Pharmacia) column (1·0 × 8·5 cm) equilibrated with PBS, pH 7·2. The column was washed with PBS and the absorbed material eluted with 0·05M α-methyl-D-glucoside in PBS. Most of the activity was recovered in the bound material, which again was dialysed and concentrated.

For further purification 2 different methods were used.

(A) In order to obtain peak distinction in gel filtrations (in spite of low protein content of the fractions at that stage), the antigen was labelled with $^{125}$iodine and the protein fraction, after passing a Sephadex G 15 gel (cf. Labelling) was layered on to a Sephadex G 200 column (0·9 × 30 cm) equilibrated and eluted with PBS, pH 7·2. The elution profile showed 2 $^{125}$I-peaks, the first appearing shortly in front of human IgG and the second shortly after human albumin.

(B) By the second method, the active fraction of the Con A column was applied to a column of wheat-germ lectin (WGL) coupled to Sepharose 6MB (Pharmacia). This column (2 ml volume) was equilibrated and washed with PBS, and the absorbed material, eluted by N-acetyl-D-glucosamine (100 mM in PBS) was dialysed very carefully (in order to remove all glucosamine) and then concentrated. The glycoproteins absorbed by the wheat germ lectin appeared to possess high specific inhibitory activity in the cytotoxic assay. The preparation was iodinated and examined for uptake by antiserum in the saturation assay (Table II), or it was submitted to successive absorption on crosslinked antisera to normal human proteins and to normal human lung tissues,

TABLE II.—*Saturation Analysis Carried out with Various Antisera and $^{125}$I-labelled Antigens*

| $^{125}$I Antigen (origin and fraction type) | Antiserum* | Maximal uptake of $^{125}$I antigen (%)§ (Bound/Total†)‡ | |
|---|---|---|---|
| Pleural Effusion | | | |
| Con A-bound material | BL, not absorbed | 35 | (34; 31; 36; 38; 35; 35) |
| | absorbed | 20 | (18; 19; 21; 22) |
| | DU, absorbed | 30 | (25; 29; 30; 35) |
| | anti-normal lung tissue, not absorbed | 8 | (7; 8) |
| | anti-human foetal protein, not absorbed | 10 | (9; 11) |
| | anti-CLL-cells, not absorbed | 13 | (10; 17; 19; 16) |
| | anti-ALL-cells, not absorbed | 3 | (3; 3) |
| | anti-normal human lymphocytes, not absorbed | 3 | (3; 3) |
| Sephadex G 200 | | | |
| Fraction: 1·5–1·7 × 10⁵d | BL, absorbed | 25 | (21; 24; 25; 29) |
| | DU, absorbed | 70 | (69; 70; 75; 68; 69; 76) |
| $<6·5 \times 10^4$d | BL, absorbed | 2 | (2; 2) |
| | DU, absorbed | 8 | (8; 8) |
| Wheat germ lectin-bound material | BL, absorbed | 15 | (14; 13; 18; 19) |
| | DU, absorbed | 55 | (55; 54; 52; 53) |
| Squamous-cell carcinoma (cells) | | | |
| Con A-bound material | BL, absorbed | 15 | (13; 15; 18; 14) |
| | DU, absorbed | 25 | (26; 28; 24; 23) |
| Material not bound | BL, absorbed | 0 | (0; 0) |
| | DU, absorbed | 0 | (0; 0) |
| E14 (cultured cells) | | | |
| Con A-bound material | BL, absorbed | 12 | (12; 10; 14; 13) |
| | DU, absorbed | 20 | (19; 20; 23; 21) |

* Antisera were titrated in 5-fold dilutions from 1 : 20 to 1 : 62,500. Maximal uptake occurred at dilutions 1 : 20–1 : 100.

† In most cases the total was 20,000 ct/min. In case of the G 200 fractions it was 6000 ct/min.

‡ Background of 6–10% subtracted before calculation.

§ Mean values of duplicates of 1–3 tests (first column), and values of single tubes (in brackets).

and then tested for inhibitory activity. The protein recovery in the fraction which was bound to the WGL-column was about 0·1% of the protein in the starting material, and this was further reduced (electrophoretic evidence not shown in this paper) by absorption on crosslinked antisera to proteins of normal human serum and lung.

*Saturation Analysis*

Fractions which proved to be good inhibitors in the cytotoxic system, along with non-inhibiting control fractions, were labelled with $^{125}I$ and tested in a saturation-type radioimmunoassay to provide additional evidence for their specificity by being selectively taken up by the specific antiserum BL. It was, furthermore, investigated whether the BL antiserum (raised by tumour cells) and the DU antiserum (raised by pleural-effusion fractions) would exhibit similar specific properties in this test, and whether crossreactions would occur between both antisera and fractions from pleural effusions, tumour-cell extracts and tissue-culture cell extracts.

It may be gathered from Table II that the labelled antigens were taken up by both antisera, BL and DU, the antiserum DU being, generally, more avid than antiserum BL. It will also be noted that the antigen uptake was selective. Unspecific antisera reacted much less with the antigen preparations, in particular the antiserum specific for normal human lymphocytes did not bind antigen at all, although it exhibited a good cytotoxic titre with normal lymphocytes (unpublished data). Besides, control preparations, *e.g.* the low-molecular-weight component obtained from the Sephadex G 200 column, and the material which was recovered in the void volume of the Con A-columns, both did not attach themselves to either antiserum. There was also a wide crossreaction between the antigens isolated from squamous-cell carcinoma tissue, pleural effusions, and the E14 culture cells and both antisera. No evidence was found for a stronger uptake of antigens from tumour cells by the BL antiserum as compared with the DU antiserum. These results thus appear to prove that the pleural effusions contain the same antigens as occur on, and can be extracted from, squamous-cell carcinoma cells. They also confirm the specificity of the $^{51}Cr$ test by showing that the uptake of labelled antigens corresponds principally to their inhibitory capacity.

### DISCUSSION

The BL antiserum (produced with squamous-cell carcinoma cells of a patient) after exhaustive absorption on normal human serum proteins and normal lung tissue, still exhibited a cytolytic effect on the E14 target cells, which could be selectively inhibited by certain isolated and purified materials. These inhibiting substances may, therefore, contain specific antigens, associated with squamous-cell carcinoma cells, which do not occur in sera and lung tissues of normal individuals (or only on very low levels).

Biochemically, the antigen would appear to be a glycoprotein with N-acetyl-glucosamine groups, which are the sugar groups specifically bound by wheat-germ lectin. To judge by elution profiles from gel-filtration columns, the molecular weight of the most purified fractions was slightly higher than that of IgG (*i.e.* $\sim 1{\cdot}7 \times 10^5$ dalton). After passing the WGL-column the specific inhibitory activity of the antigen preparation had increased considerably, an observation perhaps due to an effect similar to that described by Clemetson *et al.* (1976) who were able to separate normal transplantation antigens from tumour-specific antigens in extracts from a murine mastocytoma on columns of wheat-germ lectin.

During the course of the separation procedure, it was observed that, apart from fractions containing most of the inhibitory activity, some others of different constitution exhibited small inhibitory effects. This may be due to imperfect separation, or to different molecules carrying the same active groups. It may also indicate genuinely dissimilar, weakly crossreacting substances, and confirm the existence of various distinct TAA-activities which were recently reported by Veltri *et al.* (1977).

The molecular weight found for the described isolated antigen is close to that of carcinoembryonic antigen (CEA) (Tillack *et al.*, 1974). But, by radioimmunoassay, kindly performed by Mr B. Chaput (Hoffman La Roche, Vienna) several crude and purified fractions of the present series were all negative for CEA. The presence of $\alpha$-foetoprotein and of HLA antigens can also be ruled out, because of their respective molecular weights, and so may the involvement of blood-group antigens (mol. wt. 2–3 $\times 10^5$). Finally, contamination by bacterial antigens from pleural infections could not be detected in the antigen fractions by an antiserum raised to a wide bacterial spectrum. Some caution, concerning possible crossreactions of antigens from certain other tumours, appears to be, nevertheless, appropriate. Like the anti-CLL serum that shows a relatively high antigen uptake in the saturation assay, the BL antiserum produced a relatively high cytotoxic titre with Ewing sarcoma cells. Studies of crossreactions of the antisera with such "unrelated" antigens, and with "related" antigens such as TAAs from lung cancers of different histopathological origin, as well as the suitability of the isolated antigen as a diagnostic marker for lung cancer in clinical trials are now in progress.

I wish to express my thanks to Professor H. Wrba for generously providing the facilities for these experiments, and to Drs M. Micksche and W. Rella for co-operating and supplying clinical samples. The technical assistance of H. Bauer and the preparation of tumour and E14 extracts by E. Sandor are appreciated.

### REFERENCES

CLEMETSON, K. J., BERTSCHMANN, M., WIDMER, S. & LÜSCHER, E. F. (1976) Water-Soluble P-815 Mastocytoma Membrane Antigens: Separation of Tumour-associated Antigens from Histocompatibility Antigens. *Immunochemistry*, **13**, 383.

FISCHER, P. & VETTERLEIN, M. (1977) Establishment and Cytogenetic Analysis of a Cell Line Derived from a Human Epithelioma of the Lung. *Oncology*, **34**, 206.

FROST, M. J., ROGERS, G. T. & BAGSHAWE, K. D. (1975) Extraction and Preliminary Characterization of a Human Bronchogenic Carcinoma Antigen. *Br. J. Cancer*, **31**, 379.

GRANLUND, D. J. & RITTS, R. E. (1976) Soluble Proteins of Human Bronchogenic Carcinomas. *Mayo Clin. Proc.*, **51**, 19.

HOLLINSHEAD, A. C., STEWART, T. H. M. & HERBERMAN, R. B. (1974) Delayed-Hypersensitivity Reactions to Soluble Membrane Antigens of Human Malignant Lung Cells. *J. natn. Cancer Inst.*, **52**, 327.

HUNTER, W. M. (1974) Preparation and Assessment of Radioactive Tracers. *Br. med. Bull.*, **30**, 18.

KELLY, B. & LEVY, J. G. (1977) Evidence for a Common Tumour-associated Antigen in Extractions of Human Bronchogenic Carcinoma. *Br. J. Cancer*, **35**, 828.

MCCOY, J. L., JEROME, L. F., CANNON, G. B., WEESE, J. L. & HERBERMAN, R. B. (1977) Reactivity of Lung Cancer Patients in Leukocyte Migration Inhibition Assay to 3M Potassium Chloride Extracts of Fresh Tumour and Tissue-Cultured Cells Derived from Lung Cancer. *J. natn. Cancer Inst.*, **59**, 1413.

TILLACK, T., ROSAI, J. & VERVYNCK, D. J. (1974) Immunologic Studies of Glycoproteins Isolated from Cell Membranes of Human Lung Carcinoma. *J. natn. Cancer Inst.*, **52**, 1059.

VELTRI, R. W., MENGOLI, H. F., MAXIM, P. E., WESTFALL, S. H., GOPO, J. M., CHAOG-WEI HUANG & SPRINKLE, P. M. (1977) Isolation and Identification of Human Lung Tumour-associated Antigens. *Cancer Res.*, **37**, 1313.

WOLF, A. (1977) Antisera als Hilfsmittel in der Krebsforschung. *Öst. Ärzteztg.*, **32**, 1209.

WOLF, A. & STEELE, K. (1975) Separation of a Tumour Specific Transplantation-type Antigen from the Ascitic Fluid of Mice Bearing a Syngeneic Lymphoma. *Br. J. Cancer*, **31**, 684.

WOLF, A., STEELE, K. A. & ALEXANDER, P. (1976) Estimation in Sera by Radioimmunoassay of a Specific Membrane Antigen Associated with a Murine Lymphoma. *Br. J. Cancer*, **33**, 144.